# Effects of gut microbiota on cognitive impairment in Parkinson’s disease: a comprehensive Mendelian randomization and case–control study

**DOI:** 10.3389/fmicb.2025.1620449

**Published:** 2025-08-25

**Authors:** Yukun Feng, Qi Chang, Hao Zhou, Wei Zhang, Ling Xie, Xueyang Deng, Tao Chen, Weiguo Liu

**Affiliations:** ^1^Department of Neurology, The Affiliated Brain Hospital of Nanjing Medical University, Nanjing, China; ^2^Department of Neurology, Hainan General Hospital, Hainan Affiliated Hospital of Hainan Medical University, Haikou, China; ^3^Department of Neurology, The Affiliated Jiangning Hospital of Nanjing Medical University, Nanjing, China; ^4^Department of Pharmacology of Chinese Material Medical, China Pharmaceutical University, Nanjing, China; ^5^State Key Laboratory of Natural Medicines, Jiangsu Key Laboratory of Carcinogenesis and Intervention, China Pharmaceutical University, Nanjing, China; ^6^Hainan Provincial Bureau of Disease Prevention and Control, Haikou, China

**Keywords:** gut microbiota, Parkinson dementia disease, metabolites, causal effect, Mendelian randomization

## Abstract

**Background:**

Increasing evidence suggests a potential role of the gut microbiota in Parkinson’s disease (PD). However, the relationship between the gut microbiome (GM) and PD dementia (PDD) remains debated, with their causal effects and underlying mechanisms not yet fully understood.

**Methods:**

Utilizing data from large-scale genome-wide association studies (GWASs), this study applied bidirectional and mediating Mendelian randomization (MR) to investigate the causal relationship and underlying mechanisms between the GM and PDD. In our analysis, inverse-variance weighting (IVW) was used as the primary method. Clinical validation was performed using metagenomic sequencing and bioinformatic analysis. The relationships between the GM and PDD were visualized using receiver operating characteristic (ROC) curves, confusion matrices, and correlation analyses.

**Results:**

Our study revealed a significant causal impact of five GM genera, 10 metabolites, two metabolite ratios, and 22 immune cells on PDD. Notably, the maltose to sucrose ratio was identified as a mediator of the positive causal effect of *Subdoligranulum* on PDD, with a mediation value of 13.2%. The clinical samples confirmed the efficacy of *Subdoligranulum* sp. in distinguishing patients with PDD from normal controls (area under the curve (AUC) = 0.80, 95% CI: 0.674–0.924). In addition, correlation analysis revealed a potential negative association between *Subdoligranulum* abundance and the Mini-Mental State Examination (MMSE) scores (r = −0.316, *p* = 0.006). Finally, bioinformatic analysis suggested that *Subdoligranulum* may influence PDD risk through the regulation of starch and sucrose metabolism pathways.

**Conclusion:**

Our study confirms the potential role of *Subdoligranulum* in PDD progression, potentially mediated through starch and sucrose metabolism. These findings highlight the importance of the gut–brain axis in PDD and may provide insights into targeted interventions for PDD.

## Introduction

Parkinson’s disease (PD) is a progressive neurodegenerative disorder characterized by both motor and non-motor symptoms, and it is projected to affect more than 12 million patients globally by 2040 (Dorsey et al., 2018). Cognitive impairment (CI), a common non-motor symptom, can occur in the early stages, with approximately 80% of patients eventually progressing to dementia over the course of the disease, resulting in increased mortality and disability (Buter et al., 2008; Fengler et al., 2017; Hely et al., 2008). Moreover, no effective treatment strategies are currently available to stop or reduce cognitive decline (Weintraub et al., 2022). Therefore, early diagnosis and intervention are vital for the cognitive management of PD. Identifying biomarkers that can detect individuals at high risk of PD-associated cognitive impairment (PD-CI) and in the early stage of cognitive decline is essential. However, the underlying mechanisms through which patients with PD experience cognitive dysfunction have not been elucidated.

Constipation can occur at all stages of PD, even prior to the onset of motor symptoms (Braak et al., 2006). The composition of the microbiota residing in the intestine may change during the early phase of PD (Sun and Shen, 2018). Recently, the gut microbiome (GM) has been found to play a critical role in PD through the microbiome–gut–brain axis, a finding that has been demonstrated in both mouse and human models (Qian et al., 2020; Sampson et al., 2016). Many researchers have explored fecal microbiota transplantation as a potential treatment for PD, and this approach could be promising (Cheng et al., 2023; Sun et al., 2018; Zhao et al., 2021). Moreover, current scholars have performed observational studies on the dysbiosis of the GM in patients with PD-associated cognitive impairment (PD-CI), although the correlation between the GM and PD-CI is controversial (Aho et al., 2024; Ren et al., 2020). One possible reason for this is the susceptibility of the GM to environmental confounding factors, which can lead to inconsistent results. Randomized controlled trials (RCTs), the gold standard for studying causal relationships, are prone to limitations due to cost, logistical challenges, and potential biases (Ning et al., 2024). Therefore, previous studies have established a causal link between the gut microbiota and PD-CI using Mendelian randomization (MR) analysis to avoid the impact of confounding variables and reverse causality (Fu et al., 2024; Ji et al., 2024). In MR analysis, single-nucleotide polymorphisms (SNPs) from genome-wide association studies (GWASs) are used as instrumental variables (IVs).

The GM significantly influences key metabolic and immune processes, including host immunity, intestinal endocrine function, and intestinal permeability, which subsequently contribute to the initiation and progression of various diseases (Nakandalage et al., 2023). An increasing number of studies have shown that GM metabolites may affect the brain through the bloodstream or the vagus nerve to regulate cognitive behavior (Dogra et al., 2022; Houser and Tansey, 2017). In addition, the GM plays an important role in the regulation of the immune cell response. Inflammation, as a risk factor for cognitive impairment, has been well documented (Fung, 2020). The GM can trigger an immune cell-mediated cytokine response and further influence neuroinflammation in memory-related brain regions (Walker et al., 2019). Immune cells and GM metabolites potentially act as mediating factors in the pathway from the GM to PD-CI. However, the causal relationships among the GM, blood metabolites or immune cells, and PD-CI have not yet been clarified.

In this study, we used a bidirectional MR method to explore the causal relationships among the GM, immune cells, GM metabolites, and PD dementia (PDD). Furthermore, the relationship between five GM genera and PD-CI was evaluated using sequencing data from clinical samples. Ultimately, we identified the potential role of GM metabolites as mediators via MR analysis and a case–control study. Our findings provide a theoretical basis for understanding the mechanisms underlying PD-CI through the microbiome–gut–brain axis, as well as for the early screening and prevention of this disease.

## Materials and methods

### Mendelian randomization

#### Study design

**Figure 1:**
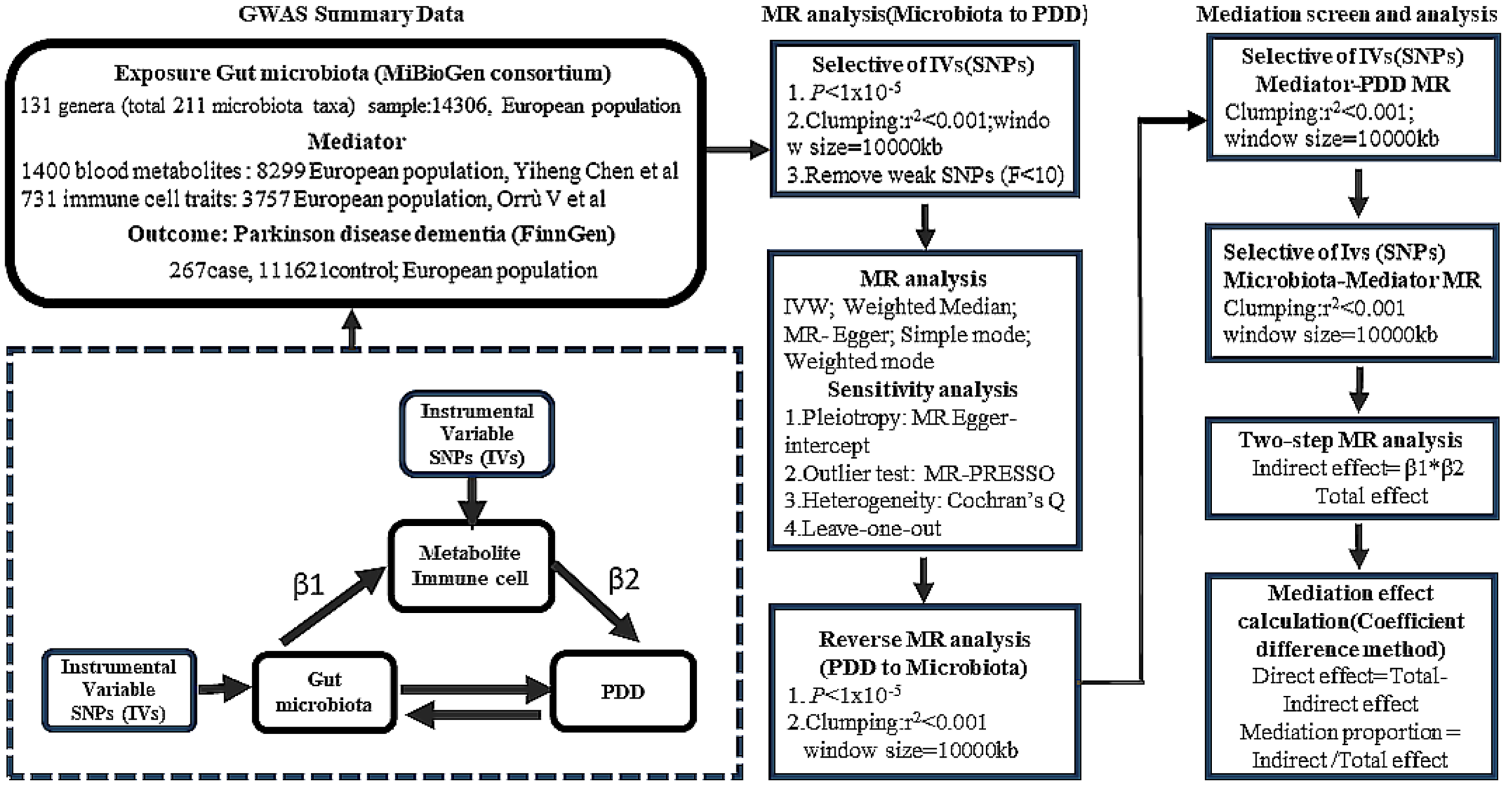
Mendelian randomization (MR) flowchart.

We employed two-sample MR to explore the potential causal relationships between the GM and PDD. To further elucidate the role of the GM in cognitive decline in PD, two-step MR analysis was performed to strengthen our understanding of the mediatory mechanisms involved. The design of our study is detailed in Figure 1.

#### Data sources

The GWAS data for the GM were obtained from the MiBioGen consortium, based on the genomic statistical research conducted by Kurilshikov et al., which included genome-wide genotype and 16S fecal microbiome data from 18,340 individuals of primary European ancestry (24 cohorts) (Kurilshikov et al., 2021).^1^ The GWAS summary data contained a total of 211 gut microbiota taxa (131 genera). In addition, we extracted summary data on mediation factors from the most extensive and up-to-date GWAS catalog, including 1,400 metabolites and 731 immune cell traits from individuals of European descent (Chen et al., 2023; Orru et al., 2020).^2^ For PDD, GWAS statistics were derived from the FinnGen study program and included 267 cases and 216,628 controls.^3^ The diagnostic criteria for PDD were based on the G20 classification according to the ICD-10 criteria.

A secondary analysis using data from publicly available databases was conducted in the present study. Ethical approval was obtained for the original GWASs. In addition, we did not include individual-level data.

#### Instrumental variable selection

In this study, SNPs associated with GM, blood metabolites, immune cells, and PDD were selected as IVs. Initially, we employed an appropriate threshold (*p* < 1 × 10^−5^) to acquire a larger number of IVs for preliminary screening. Then, we rigorously excluded SNPs exhibiting linkage disequilibrium (LD) (r2 < 0.01, window size > 10,000 kb). To determine whether the identified IVs were strongly associated, F statistics were calculated. Generally, SNPs with F-statistics less than 10 were considered weak instrumental variables and excluded from the analysis.

### Statistical analysis

#### MR analysis (primary analysis)

To assess the causal relationship between the GM and PDD, five methods were used: inverse-variance weighting (IVW), weighted median (WM), MR-Egger, simple mode, and weighted mode methods. We selected the IVW method as the primary analytical approach to ascertain the validity of all the genetic instruments used. The MR results were expressed as odds ratios (ORs) and 95% confidence intervals (CIs). A causal relationship was considered if the IVW method yielded significant results (*p < 0.05*) and if the directions of the IVW and other methods were consistent.

#### Bidirectional causality analysis

We evaluated the bidirectional causation effects between the GM and PDD using reverse MR analysis. PDD was used as the “exposure,” and the GM related to PDD was used as the “outcome” (Figure 1). We selected SNPs significantly associated with PDD (*p* < 1 × 10^−5^) as IVs.

#### Mediation analysis

We analyzed the mediating effect of blood metabolites or immune cells on the causal relationship between the GM and PDD using two-step MR analysis. The proportion of the indirect effect mediated by blood metabolites or immune cells (β1 × β2) to the total effect was estimated, where β1 represents the impact of the GM on blood metabolites or immune cells and β2 represents the impact of blood metabolites or immune cell amino acids on PDD. Effect estimates were obtained using two-sample MR analysis.

#### Sensitivity analysis

The heterogeneity of each SNP was evaluated using Cochran’s Q test. We performed Mendelian randomization pleiotropy residual sum and outlier (MR-PRESSO) analyses to detect significant SNP outliers with pleiotropic effects and to correct estimates by removing the outliers. In addition, MR-Egger regression was used to assess the potential horizontal pleiotropy effect through its intercept test. Finally, we employed leave-one-out analysis to determine the impact of each individual genetic variant on the overall MR estimate. All the statistical analyses were performed using the R software (v4.3.3). The “Two SampleMR,” “MRPRESSO,” “ggplot2,” and “circlize” R packages were used for the MR study and data visualization.

### Case–control study

#### Participant recruitment

This case–control study was conducted at Nanjing Brain Hospital of Nanjing Medical University from June 2024 to January 2025. Patients with PD with normal cognition (PD-NC) and patients with PDD were selected from the Department of Neurology and were diagnosed with idiopathic PD according to the UK Brain Bank criteria (Daniel and Lees, 1993). The exclusion criteria for patients were as follows: (i) atypical or secondary parkinsonism; (ii) serious illness (e.g., heart failure or malignancy); (iii) inflammatory gastrointestinal disease and history of gastrointestinal surgery; (iv) hematological or autoimmune disease, or use of immunosuppressive agents within the past 3 months; and (v) antibiotic use within 3 months prior to sample collection. Patients with PDD were identified using education-specific cutoff points on the Mini-Mental State Examination (MMSE) for elderly Chinese individuals: MMSE score ≤17 for illiterate individuals, ≤20 for those with 1–6 years of education, and ≤ 24 for those with 7 or more years of education (Li et al., 2016). Healthy controls (HCs) exhibited no disease symptoms and met none of the exclusion criteria. Simultaneously, age, sex, education, and body mass index (BMI) were recorded for all participants. The clinical characteristics of the patients with PD were obtained through face-to-face interviews, including the Hoehn and Yahr (H-Y) stage, the unified Parkinson’s disease rating scale III (UPDRS-III) score, disease duration, Parkinson’s Disease Questionnaire-39 (PDQ39) score, levodopa equivalent dose (LED), and Mini-Mental State Examination (MMSE) score. This study protocol was approved by the Ethics Committee of Nanjing Brain Hospital, Nanjing Medical University (no. 2023-KY056-01), and all participants signed an informed consent form.

#### Fecal sample storage, DNA extraction, and shotgun sequencing

All participants’ fecal samples were frozen and stored at −80°C in a refrigerator immediately after collection in containers with 2 mL of professional stool DNA preservation solution (Dongyuan Yikang Pharmaceutical Technology Co., Ltd., Beijing, China). Total fecal DNA was extracted using a MagPure Soil DNA KF Kit (Magen Biotechnology Co., Ltd., Guangzhou, China) according to the manufacturer’s instructions. All DNA extraction procedures were performed in a Class II biological safety cabinet. The concentration of genomic DNA in each sample was quantified using a NanoDrop 2000 spectrophotometer (Thermo Scientific, United States). The extracted microbial DNA was processed to construct metagenome shotgun sequencing libraries according to the manufacturer’s instructions (Illumina, United States). Each library was sequenced using the Illumina NovaSeq platform at Biomiao Biotechnology Corporation (Beijing, China). Briefly, the DNA samples were randomly sheared into fragments of approximately 350 bp using a Covaris S2020 ultrasonic crusher (Gene Company, United States). The library was constructed through a series of steps, including end repair, adapter attachment, library amplification, and purification. The length of the library was assessed using an Agilent 2,100 Bioanalyzer (Agilent Technologies, United States), and the library concentration was quantified using a Qubit® 3.0 fluorometer (Life Technologies, United States).

### Statistical analysis

The DIAMOND (Buchfink et al., 2015) software was used to compare the representative sequences (amino acid sequences) of the redundant gene set with the NCBI NR, Kyoto Encyclopedia of Genes and Genomes (KEGG), and Gene Ontology (GO) databases. Functional annotations were assigned a threshold of e < 1e-5, and the protein with the highest sequence similarity was selected. The gene sets were compared with the carbohydrate-active enzymes (CAZymes) database using the hmmscan tool (v3.1b2) to obtain information on the carbohydrate-active enzymes corresponding to the gene. Then, carbohydrate activity was calculated using the sum of the gene abundances corresponding to the carbohydrate-active enzyme abundance. Species annotations were obtained from the NR database’s taxonomic information, and gene abundances were summed to calculate genus-and species-level abundances. According to MR analysis, the relative abundances of the five GM genera were extracted from genus-level abundance data, and promising genera were further analyzed at the species level. Differences in GM and carbohydrate-active enzyme abundances among the patients with PD-NC, patients with PDD, and HCs were subsequently analyzed using the Kruskal–Wallis test. For comparisons of clinical and demographic characteristics across the multiple groups, statistical analyses were performed using one-way ANOVA, the chi-squared test, the Mann–Whitney U test, or the Kruskal–Wallis test, as appropriate. Multiple comparisons were adjusted using the Bonferroni correction, with *p* < 0.05 considered statistically significant. Statistical power was also assessed because the sample size was relatively small^4^ (Cohen, 1992; Houle et al., 2005). The area under the receiver operating characteristic curve (AUC-ROC) was used to detect the distinguishing ability of biomarkers. The Pearson correlation coefficient was used to describe the correlation between the measurement data. All statistical analyses were performed using R (version 4.3.3, the R Project for Statistical Computing) and SPSS (version 24.0, SPSS Inc., United States).

## Results

### Causal effects of the gut microbiota on PDD

Initially, we identified a total of 1,531 SNPs that were significantly associated with the GM at the genus level by screening at a threshold of *p* < 1 × 10^−5^, and we excluded SNPs with linkage disequilibrium (LD), as detailed in Supplementary Table 1. All SNPs analyzed had *F*-values greater than 10 (Supplementary Table 1). Our analysis revealed that the five GM genera were significantly related to PDD (Supplementary Table 2). Specifically, information about 65 SNPs for the five GM genera is presented in Supplementary Table 2.

**Figure 2:**
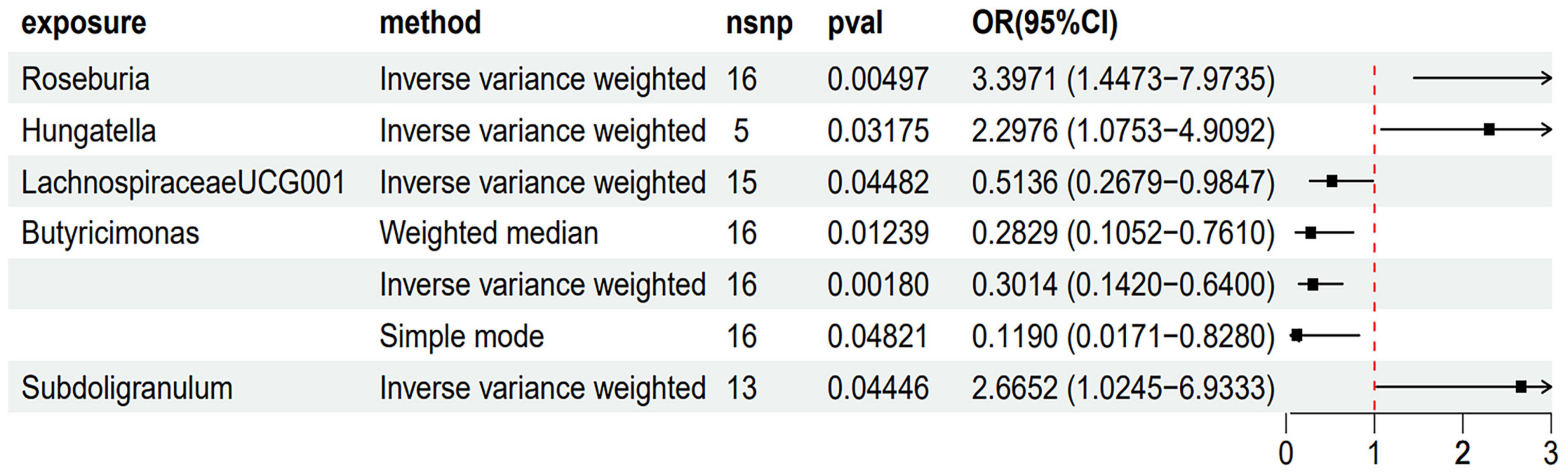
MR analysis of the causal effects of the gut microbiota on Parkinson’s disease dementia (PDD). This forest plot displays the associations between specific gut microbiota taxa and PDD risk. Each point represents the odds ratio (OR) for a single taxon, showing the strength and direction of the association. Horizontal bars represent the confidence intervals (CIs), indicating the precision of the OR estimate. Points positioned to the left of the vertical reference line (OR < 1) suggest a protective effect against PDD, while points to the right (OR > 1) indicate a potential risk. *p*-values assess the statistical significance of each association.

As shown in Figure 2 and Supplementary Table 3, the results of the MR analysis using the IVW method revealed that *Roseburia* (odds ratio (OR) = 3.3971, 95% confidence interval (CI) = 1.4473–7.9735, *p* = 0.00497), *Hungatella* (OR = 2.2976, 95% CI = 1.0753–4.9092, *p* = 0.03175), and *Subdoligranulum* (OR = 2.6652, 95% CI = 1.0245–6.9333, *p* = 0.0446) were potential risk factors, indicating a promoting role in the onset of PDD. Conversely, *LachnospiraceaeUCG001* (OR = 0.5136, 95% CI = 0.2679–0.9847, *p* = 0.04482) was related to a reduced risk of PDD. *Butyricimonas* (OR = 0.3014, 95% CI = 0.1420–0.6400, *p* = 0.00180) also showed a similar protective trend against PDD. This relationship was further validated using both WM and simple mode methods. Furthermore, the results of the reverse MR analysis did not indicate any significant causal effects of PDD on the five microbiota genera, as reported in Supplementary Table 3.

### Clinical validation of the gut microbiota

**Figure 3:**
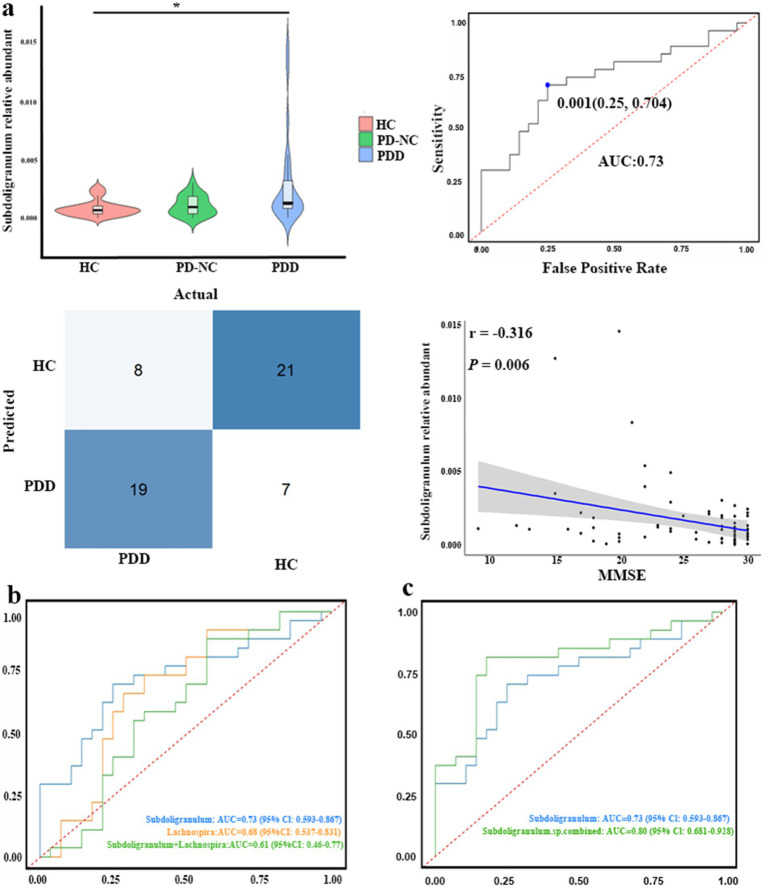
Clinical validation of the gut microbiota genera identified using MR analysis. **(a)** Top row, left: differences in *Subdoligranulum* abundance among the healthy controls (HC), patients with PD with normal cognition (PD-NC), and patients with PDD (Kruskal–Wallis test). Top row, right: receiver operating characteristic (ROC) curve assessing the ability of *Subdoligranulum* abundance to discriminate patients with PDD from HC. Bottom row, right: confusion matrix illustrating *Subdoligranulum*’s classification performance (PDD vs. HCs). Bottom Row, right: scatter plot showing the association between *Subdoligranulum* abundance and the Mini-Mental State Examination (MMSE) scores. **(b)** ROC curves comparing the diagnostic performance of *Subdoligranulum, Lachnospira*, and their combination (*Subdoligranulum* + *Lachnospira*) for discriminating patients with PDD from HCs. **(c)** ROC curves comparing the diagnostic performance of genus-level *Subdoligranulum* abundance versus the combined abundance of multiple differentially abundant *Subdoligranulum* species (*Subdoligranulum* sp. combined) for discriminating patients with PDD from HCs. *p < 0.05.

In our case–control study, fecal samples were collected from 75 participants, including 27 patients with PDD, 20 patients with PD-NC, and 28 HCs. The baseline demographic and clinical characteristics of all participants were compared and are summarized in Table 1. Following the confirmation of the causal association direction through MR analysis, we proceeded to evaluate the five GM genera identified in the case–control study. We extracted the relative abundances of *Roseburia*, *Hungatella*, *Subdoligranulum*, *Lachnospira,* and *Butyricimonas* according to the corresponding groups and performed comparative analyses. *Hungatella* was not significantly different between the corresponding groups (*p* = 0.093). Compared to the patients with PDD, the HCs presented a significantly greater relative abundance of *Subdoligranulum* (*p* = 0.004, Bonferroni-corrected *p* = 0.011) (Figure 3a). The area under the curve (AUC) for *Subdoligranulum* was 0.73 (95% CI: 0.59–0.87), with an optimal cutoff value of 0.001 determined from the receiver operating characteristic (ROC) curve (Figure 3a). The confusion matrix evaluation revealed that the sensitivity, specificity, and accuracy of this biomarker were 0.70, 0.75, and 0.72, respectively (Figure 3a). Furthermore, the relative abundance of *Subdoligranulum* was negatively correlated with the MMSE score (r = −0.316, *p* = 0.006) (Figure 3a). In contrast, the relative abundance of *Lachnospira* was significantly lower in the HCs than in the patients with PDD (*p* = 0.023, Bonferroni-corrected *p* = 0.069) (Supplementary Figure 2a). The AUC for *Lachnospira* was 0.68 (95% CI: 0.54–0.83), with an optimal cutoff value of 0.007 (Supplementary Figure 2a). The sensitivity, specificity, and accuracy were 0.74, 0.64, and 0.69, respectively, as determined from the confusion matrix evaluation (Supplementary Figure 2a). In addition, the abundance of *Lachnospira* was significantly positively correlated with the MMSE scores (r = 0.271, *p* = 0.019) (Supplementary Figure 2a). The combined ROC curve analysis demonstrated that *Subdoligranulum* exhibited promising performance in distinguishing patients with PDD from HCs, whereas *Lachnospira* showed limited discriminative ability (Figure 3b). We extracted significantly more species-level data on *Subdoligranulum* from the patients with PDD compared to the HCs and conducted a combined ROC curve analysis to improve the diagnostic accuracy. The AUC for the seven *Subdoligranulum* species (*Subdoligranulum* sp.4_3_54A_2_FAA, AF14-43, AM 16-9, AM23-21 AC, OF01-18, TF05-17 AC, and CAG:314) was 0.80 (95% CI, 0.681-0.928), which demonstrated strong potential as biomarkers for distinguishing patients with PDD from HCs, outperforming the genus-level *Subdoligranulum* analysis (Figure 3c; Supplementary Table 7). These findings highlight the potential of *Subdoligranulum* as a microbial biomarker for distinguishing patients with PDD from HCs, as validated through MR analysis and case–control studies. In contrast, the results for *Roseburia* and *Butyricimonas* were inconsistent with the MR results. The HCs displayed a significantly greater relative abundance of *Roseburia* compared to the patients with PD (*p* = 0.011, Bonferroni-corrected *p* = 0.033) and PDD (*p* = 0.017, Bonferroni-corrected *p* = 0.052) (Supplementary Figure 2b). Conversely, the relative abundance of *Butyricimonas* was lower in the HCs than in the patients with PDD (*p* = 0.001, Bonferroni-corrected *p* = 0.003) (Supplementary Figure 2b).

**Table 1:** Demographic and clinical characteristics of participant.

Characteristic	HC (*n* = 28)	PD-NC (*n* = 20)	PDD (*n* = 27)	*P* value
Age, y	58.86 ± 3.73	61.15 ± 8.28	67.19 ± 7.59	<0.001^a***^
Gender (M/F)	15/13	14/6	8/19	0.02^c^
Education (y)	10.89 ± 1.97	12.30 ± 2.74	6.67 ± 5.08	<0.001^a***^
BMI (kg/m2)	24.70 ± 2.79	24.51 ± 3.08	24.80 ± 2.96	0.944^a^
Duration (y)	-	6.95 ± 3.76	7.44 ± 3.46	0.538^d^
UPDRS-III	-	33.20 ± 16.45	47.67 ± 18.19	0.005^d**^
H-Y stage	-	2.28 ± 0.73	2.63 ± 0.75	0.098^d^
PDQ39	-	29.90 ± 21.53	43.04 ± 21.24	0.016^d*^
LED (mg/day)	-	709.13 ± 293.67	599.44 ± 305.15	0.333^d^
MMSE	29.18 ± 0.90	28.50 ± 1.15	19.30 ± 4.10	<0.001^b***^

Data are means ± standard deviations; Unified Parkinson’s Disease Rating Scale, Part III, UPDRS-III; Hoehn and Yahr stage, H-Y stage; Parkinson’s Disease Questionnaire-39, PDQ39; Levodopa equivalent dose, LED; Mini-Mental State Examination, MMSE; ^a^ Age: NC vs PDD^**^, PDD vs HC^***^; ^a^Education: NC vs PDD^***^, HC vs PDD^***^; ^b^UPDRS-III: NC vs PDD^*^; ^b^MMSE score: NC vs MCI^**^, NC vs PDD^***^, MCI vs PDD^***^, MCI vs HC^***^, HC vs PDD^***^; ^b^MoCA score: NC vs MCI^***^, NC vs PDD^***^, MCI vs PDD^***^, MCI vs HC^***^, HC vs PDD^***^; a: one- ANOVA test; b: Kruskal−Wallis test; c: Chi-squared test; d: Mann–Whitney U. *p < 0.05; ^**^ *p* < 0.01; ^***^ *p* < 0.001.

### Mediation analysis

**Figure 4:**
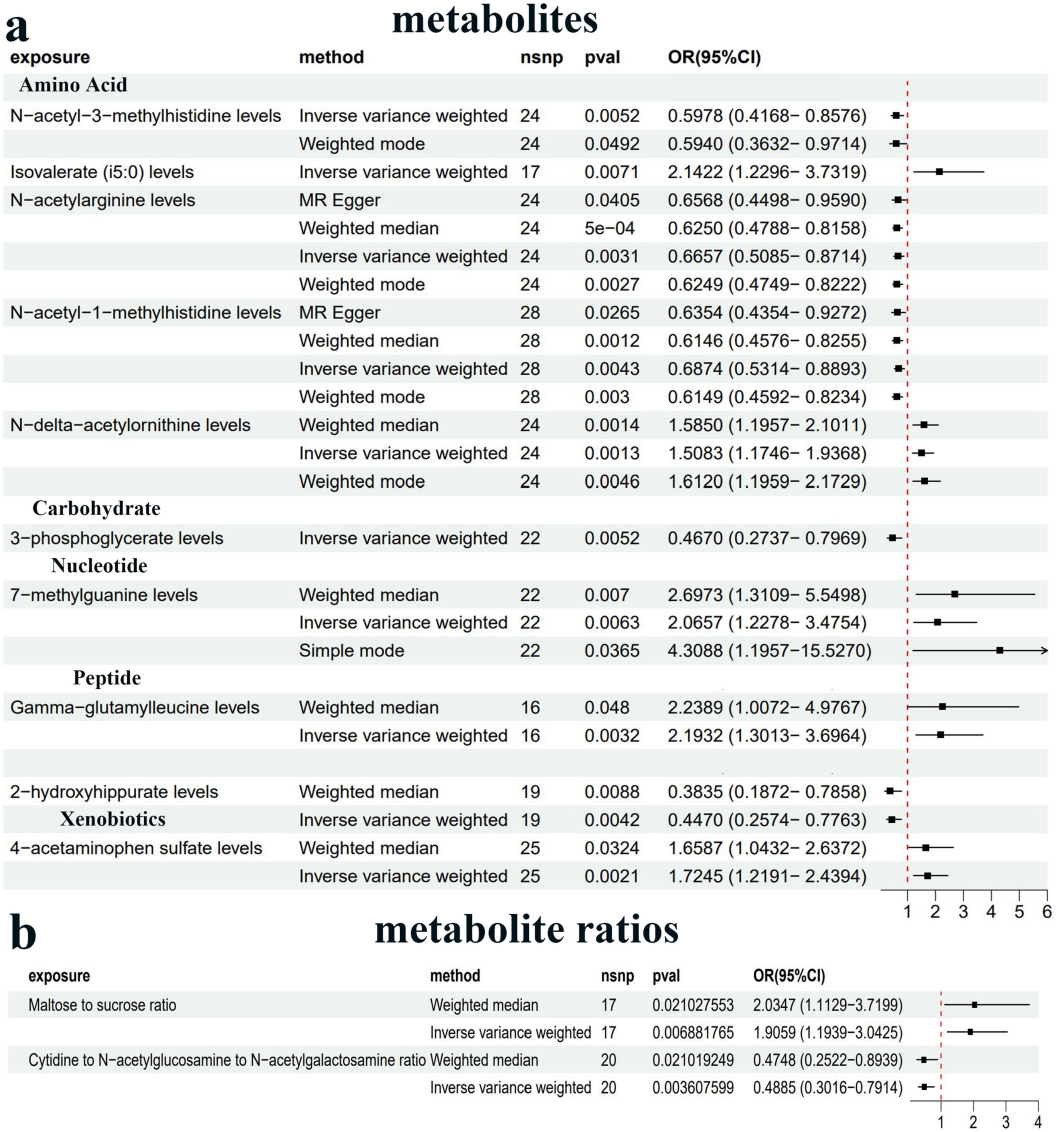
Forest plot of the causal estimates from MR analysis showing the effects of 10 metabolites **(a)** and two metabolite ratios **(b)** on PDD using the inverse-variance weighting (IVW) method, filtered *p*-values < 0.01.

**Figure 5:**
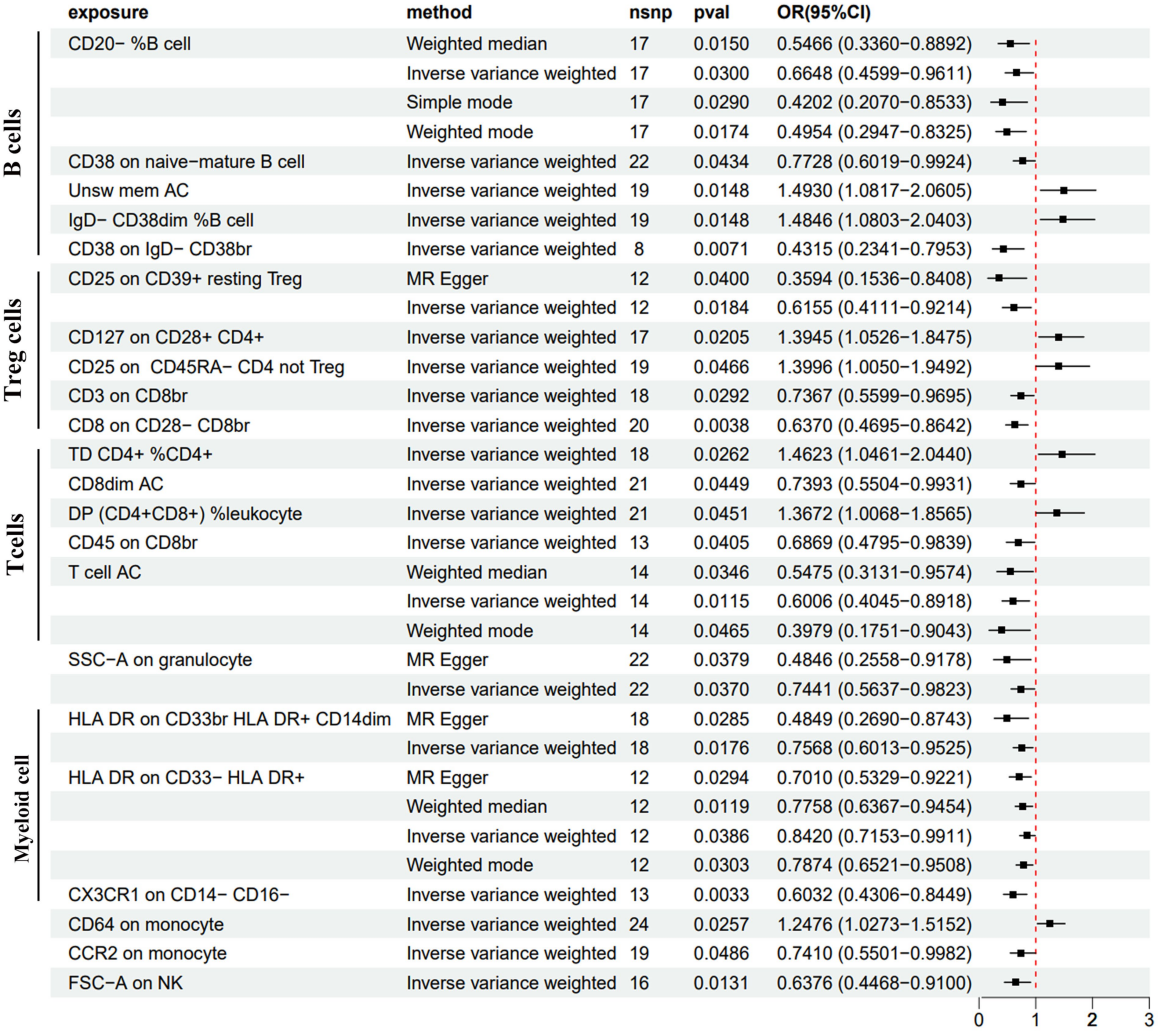
Forest plot of the causal estimates from MR analysis showing the effects of 22 immune cells on PDD using the IVW method, filtered *p*-values < 0.05.

**Figure 6:**
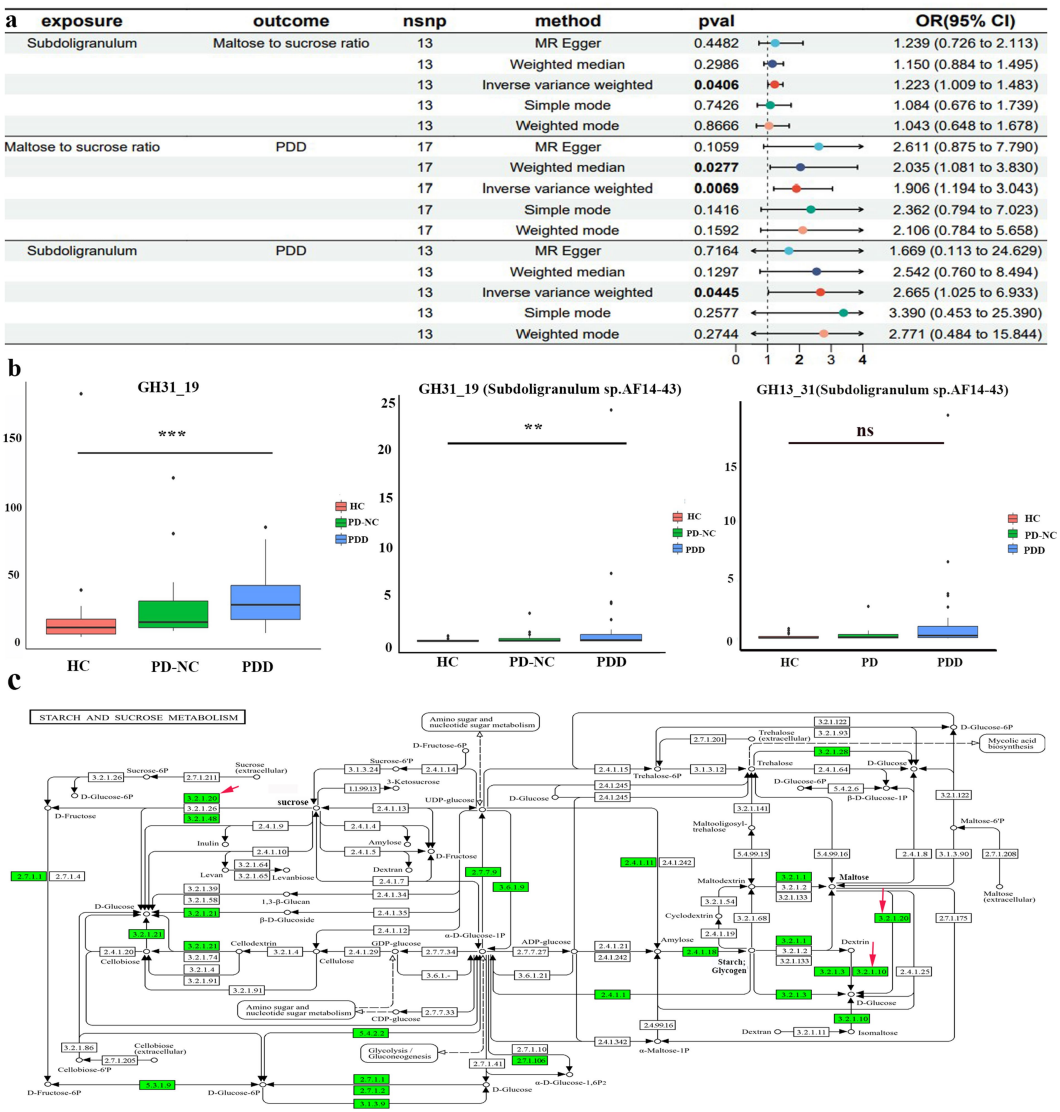
MR and clinical validation. **(a)** Forest plot of the causal estimates from mediation analysis showing the effects of the mediator on the pathway from the gut microbiota to PDD. **(b)** Differences in glycoside hydrolase family 31 (GH31_19) abundance derived from all *bacterial* species (left) and *Subdoligranulum* sp. AF14-43 (center) among the HCs, patients with PD-NC, and patients with PDD; differences in GH13_31 abundance derived from *Subdoligranulum* sp. AF14-43 (right) among the HCs, patients with PD-NC, and patients with PDD (Kruskal–Wallis test); **(c)** starch sucrose metabolic pathways map (KEGG pathway ko00500) from Kyoto Encyclopedia of Genes and Genomes (KEGG). Red arrows indicate enzymatic activities, with EC 3.2.1.20 representing *α*-glycosidase and EC 3.2.1.10 representing oligo-1,6-glucosidase. **p < 0.01 and ***p < 0.001.

We conducted MR analysis to explore the intermediary roles of immune cell traits and metabolites in the pathway from the GM to PDD. IV selection for immune cell traits and metabolites was similar to that used for the gut microbiota. Next, we applied the IVW method as the main evaluation standard, and other methods, including MR-Egger, weight mode, simple mode, and weighted median methods, served as auxiliary analysis tools. This analysis identified 10 metabolites, two metabolite ratios, and 22 immune cells (Figures 4, 5; Supplementary Table 4; Supplementary Figures 3a,b). Specifically, five metabolites (isovalerate, 7-methylguanine, gamma-glutamylleucine, 4-acetaminophen sulfate, and N-delta-acetylornithine) and one metabolite ratio (maltose to sucrose ratio) were identified as potential risk factors for PDD, whereas five additional metabolites (N-acetyl-3-methylhistidine, 2-hydroxyhippurate, 3-phosphoglycerate, N-acetylarginine, and N-acetyl-1-methylhistidine) and one metabolite (cytidine to N-acetylglucosamine/N-acetylgalactosamine) were found to exert a protective causal effect against PDD (Figure 4). Further analysis, as depicted in Figure 5, revealed that among the 22 immune cell types, 15 were significantly associated with a reduced risk of PDD, but the remaining seven types were associated with an increased risk of PDD. Notably, our MR analysis indicated that *Subdoligranulum* influences maltose and sucrose metabolism (OR = 1.223, 95% CI = 1.009–1.483, *p* = 0.0406), thereby promoting PDD development, with a mediated proportion of 13.2% (Figure 6a; Supplementary Figure 3c).

To further elucidate the biological implications of MR, fecal samples were collected from the clinical patients for metagenomic sequencing. In our case study, we observed a significant increase in the gene abundance of total glycoside hydrolase family 31 (GH31_19) (Bonferroni-corrected *p* < 0.001) and GH31_19 from *Subdoligranulum* sp. AF14-43 (Bonferroni-corrected *p* < 0.01) in the patients with PDD compared to the HCs (Figure 6b). Moreover, there was an increasing trend in the gene abundance of GH13_31 from *Subdoligranulum* sp. AF14-43 (*p* = 0.08) in the patients with PDD compared to the HCs. Functional annotations using KEGG analysis revealed that GH31_19 and GH13_31 from *Subdoligranulum* sp. AF14-43 are involved in galactose metabolism, starch and sucrose metabolism, and metabolic pathways (KO 00052, KO 00500, KO 01100). The starch and sucrose metabolism pathways include the key enzymes alpha-glucosidase (EC 3.2.1.20) and sucrase-isomaltase (EC 3.2.1.10). The identified KO symbols included malZ (encoding alpha-glucosidase [EC:3.2.1.20]) from GH31_19 and IMA and malL (both encoding oligo-1,6-glucosidase [EC:3.2.1.10]) from GH13_31 in *Subdoligranulum* sp. AF14-43. This information reveals a biological mechanism for the MR findings, suggesting that *Subdoligranulum* may influence PDD risk through the regulation of sucrose and maltose metabolism (Figure 6c).

### Sensitivity analyses

The MR-Egger regression intercept revealed no horizontal pleiotropy, with *p-*values greater than 0.05 (Supplementary Table 6). In addition, no outliers were detected using MR-PRESSO analysis (*p* > 0.05). Furthermore, the Cochran’s Q test revealed no significant heterogeneity (*p* > 0.05). No impact on the overall results was observed after the removal of any SNP in the leave-one-out analysis (Supplementary Figure 1). Therefore, the MR analysis revealed the robustness and reliability of our study.

## Discussion

In our investigation, we established significant genetic correlations between five GM genera—including *Roseburia*, *Hungatella, Subdoligranulum*, *LachnospiraceaeUCG001*, and *Butyricimonas—*and PDD. Subsequently, a case–control study validated that *Subdoligranulum* may serve as a potential biomarker for PD-CI progression. Integrated MR analysis and the case–control study revealed that *Subdoligranulum* potentially influences carbohydrate metabolism to induce Parkinson’s cognitive impairment via the gut–brain axis. These findings provide new insights into the effects of gut microbiota–metabolite interactions on the pathogenesis of PDD.

Several studies have reported alterations in GM composition among patients with cognitive impairment or PD, prompting the exploration of the GM as a potential biomarker for this disease (Varesi et al., 2022; Verhaar et al., 2021). We systematically assessed the causal relationship between intestinal bacteria and the development of PDD by integrating data from GWAS summary statistics and MR analysis. We detected a suggestive association between increased *Roseburia* abundance and increased PDD risk, as well as a link between increased *Butyricimonas* abundance and reduced PDD susceptibility, which is consistent with previous MR studies (Fu et al., 2024; Ji et al., 2024). However, our case–control studies revealed a significant reduction in *Roseburia* abundance and an increase in *Butyricimonas* abundance in the patients with PDD. Our cross-sectional findings on *Butyricimonas* abundance align with those of a Chinese PD-CI cohort study, although reduced *Butyricimonas* abundance has been reported in other cognitive impairment cohorts (Liang et al., 2022; Olazaran et al., 2015; Ren et al., 2020). Notably, previous observational studies have associated lower *Roseburia* abundance with worse progression of motor, non-motor, and cognitive functions, as well as higher levodopa doses in patients with PD (Cilia et al., 2021; Wallen et al., 2020). Similarly, a lower abundance of *Butyricimonas* correlated with worse non-motor symptoms in patients with PD (Nuzum et al., 2023). These discrepancies indicate that complex confounding factors (e.g., medication, disease severity, and symptom subtypes) may exert significant short-or long-term effects on the GM in traditional clinical studies, effects that may not be captured by genetic instruments reflecting lifetime bacterial abundance variation. Consequently, the relationships among *Roseburia*, *Butyricimonas,* and PDD should be viewed cautiously, and further investigation is needed to clarify their roles in PDD pathogenesis. In addition, this MR study revealed an association between reduced *LachnospiraceaeUCG001* abundance and reduced cognitive performance in patients with PD, which is consistent with our case–control study. Indeed, it was reported that a decreased abundance of *Lachnospiraceae CG001* was associated with cognitive decline and PD (Jiang et al., 2023; Klee et al., 2024; Proano et al., 2023). Therefore, we hypothesize that *LachnospiraceaeUCG001* could play a potential protective role against PDD progression, as supported by our results and those of previous studies. However, ROC curve analysis and confusion matrix evaluation demonstrated that the role of *Lachnospiraceae UCG001* in cognitive impairment was less prominent than that of *Subdoligranulum*. Interestingly, both our integrated case–control study and MR analysis revealed a significant association between *Subdoligranulum* and PDD development, suggesting its potential as a biomarker or therapeutic target. Nevertheless, whether *Subdoligranulum* can be considered a gut microbiota-associated factor in PD-CI remains unclear. Future animal studies are needed to elucidate its causal role in PDD pathogenesis.

The gut–brain interplay involves many pathways, one of which is mediated by active metabolites synthesized by the microbiota (Zheng et al., 2021). These metabolites, encompassing a spectrum of essential biomolecules such as amino acids, lipids, carbohydrates, and nucleotides, are pivotal to human health (Shaffer et al., 2017) and can function as neuromodulators, affecting brain function (Zheng et al., 2021). Our MR analysis identified the serum maltose-to-sucrose ratio as a mediator of the positive causal effect of *Subdoligranulum* on PDD. However, this cross-omics MR mediation analysis failed to elucidate the precise *in vivo* biological mechanisms underlying this association. To investigate potential pathways, we performed fecal metagenomic sequencing in our case–control study, profiling CAZymes and associated KEGG pathways. This analysis revealed a significant increase in *Subdoligranulum* sp. AF14-43-derived GH31_19 (alpha-glucosidase [EC:3.2.1.20]) in the patients with PDD. GH13_31 (oligo-1,6-glucosidase [EC:3.2.1.10]) levels were also elevated, although not significantly. As illustrated in Figure 6c, alpha-glucosidase hydrolyzes maltose and sucrose into glucose, whereas oligo-1,6-glucosidase converts dextrin to glucose, concurrently resulting in the accumulation of maltose. These findings provide indirect evidence that *Subdoligranulum* may promote PDD development through gut glucose metabolic dysregulation and elevated blood glucose, offering biological support that complements the MR results. This potential mechanism aligns with recognized metabolic comorbidities in PD. For example, impaired glucose tolerance is evident in moderate-to-advanced PD stages, with 58% of non-diabetic patients exhibiting systemic insulin resistance (Hogg et al., 2018; Marques et al., 2018). The proposed contributing factors include intestinal microbial disorders, autonomic nerve dysfunction, abnormal insulin signal transduction, and reduced dopaminergic receptor activity (Chen et al., 2025). These disturbances may trigger oxidative stress, a microglial inflammatory response, amyloid-*β* deposition, and pathological Tau hyperphosphorylation in brain regions associated with cognition (Chakrabarty et al., 2022; Gaspar et al., 2016; Macauley et al., 2015; Sima et al., 2009; Tomlinson and Gardiner, 2008), thereby leading to neuronal damage and mitochondrial dysfunction, which ultimately results in cognitive decline in patients with PD. In support of this finding, a Korean cohort study revealed that SGLT2 inhibitors reduce dementia and PD risk in type 2 diabetes patients, underscoring the neuroprotective potential of improving insulin resistance (Kim et al., 2024). Consequently, one possible explanation for this discovery is that *Subdoligranulum* in the gut potentially activates enzymes that hydrolyze maltose and sucrose into glucose, potentially contributing to glucose neurotoxicity and subsequent cognitive decline in patients with PD. Further animal and multi-omics studies are needed to elucidate how *Subdoligranulum* affects PDD pathology via disturbances in carbohydrate metabolism.

Our study established a compelling link between specific GM genera and PDD and identified a novel microbiota–metabolite–PDD mechanism through an integrated case–control study and MR analysis, a step previously unaccomplished. These findings support the development of adjunctive therapies (e.g., targeted dietary modifications or probiotics) alongside dopaminergic treatment to prevent cognitive decline in patients with PD-CI. Furthermore, these findings may facilitate earlier identification of at-risk patients with PD-CI, allowing timely diagnostic and therapeutic strategies to improve prognosis. However, microbiota research continues to face significant challenges due to substantial interindividual heterogeneity driven by environmental exposure, dietary habits, pharmacological treatments, and comorbidities. Therefore, future studies should prioritize multiomics approaches that integrate the gut microbiota, metabolomics, and neuroimaging within large cohorts to establish early diagnostic frameworks and effective interventions.

Although our study provides novel insights into the relationship between *Subdoligranulum* and PDD, several limitations must be acknowledged. First, our clinical sample validation relied on cross-sectional studies. Medium effect sizes were observed for group differences, yet statistical power largely remained below 0.8, indicating that the small sample size limited power (Table 2). Together, these factors limit the current strength of evidence, necessitating further validation through animal models and large cohort studies to establish robust causality. Second, the GWAS data for the GM (MiBioGen) and PDD traits (FinnGen) were derived predominantly from European populations. Although we included partial validation in an Asian cohort, this demographic bias may limit the generalizability of our findings to other ethnic populations, particularly given potential variations in gene–environment interactions across ethnicities. Therefore, future multiethnic studies are essential. Third, the selected IVs may still be susceptible to horizontal pleiotropy. Factors such as the intricate interplay among bacterial species, genetic background, lifestyle choices, and environmental conditions can profoundly shape the composition and function of the gut microbiome, resulting in a relatively small proportion of variance explained by IVs. Furthermore, the current study could not assess whether IVs are associated with confounding factors.

**Table 2:** Differences of gut microbiota and carbohydrate active enzyme abundance in case–control study.

Microbiota / Enzyme	HC (*n* = 28)	PD-NC (*n* = 20)	PDD (*n* = 27)	*P* value	*P*_FDR-corrected_ value	Power	Eta squared (η^2^_H)
*Roseburia*	0.017804 (0.0255)	0.008067 (0.0186)	0.008942 (0.0113)	0.015*	0.024*	0.622	0.088
*Hungatella*	0.000437 (0.0003)	0.000437 (0.0005)	0.000646 (0.0036)	0.093	0.093	0.304	0.038
*Lachnospira*	0.010449 (0.0176)	0.004669 (0.0116)	0.003327 (0.0085)	0.038*	0.05	0.475	0.063
*Butyricimona*	0.000964 (0.0018)	0.002711 (0.0044)	0.003960 (0.0070)	0.004**	0.016*	0.783	0.125
*Subdoligranulum*	0.000719 (0.0007)	0.000972 (0.0015)	0.001217 (0.0027)	0.013*	0.024*	0.647	0.093
GH31_19	7.34 (12.99)	11.58 (22.96)	24.77 (35.5101)	0.0004***	0.0032*	0.933	0.193
GH31_19 (*Subdoligranulum* sp. *AF14_43*)	0.079 (0.31)	0.034 (0.09)	0.156 (0.83)	0.011*	0.024*	0.667	0.097
GH13_31 (*Subdoligranulum* sp. *AF14_43*)	0.056 (0.15)	0.111 (0.35)	0.246 (1.52)	0.087	0.093	0.318	0.04

Data are median and interquartile range. Differences between three groups were assessed using Kruskal-Wallis test. Effect size is calculation according to η^2^_H, an effect size of 0.01 is small, an effect size of 0.06 is average and an effect size of 0.14 is high. ^*^p < 0.05; ^**^p < 0.01; ^***^p < 0.001.

In conclusion, our study provides novel insights into the potential causal relationships among *Subdoligranulum*, carbohydrate metabolism, and PDD. The identification of *Subdoligranulum* as a potentially promising biomarker and a therapeutic target adds to our understanding of the intricate interplay between the gut and brain in the development of PDD. These results may provide the basis for future therapeutic strategies for PDD.

## Data Availability

The dataset associated with this study is not publicly available due to confidentiality restrictions. However, the dataset can be made available upon reasonable request to the corresponding author, subject to approval and adherence to applicable regulations and conditions. Requests to access these datasets should be directed to Weiguo Liu. wgliunbh@sina.com.

## Footnotes

^1^ https://mibiogen.gcc.rug.nl

^2^ https://www.ebi.ac.uk/gwas/home

^3^ https://r5.finngen.fi/

^4^ https://www.psychometrica.de/effect_size.html

## Supplementary material

SUPPLEMENTARY FIGURE 1 **(a,b)** MR leave-one-out sensitivity for five gut microbiota on PD, *Subdoligranulum on* maltose to sucrose ratio and maltose to sucrose ratio on PDD.

SUPPLEMENTARY FIGURE 2 Clinical evaluation of *Roseburia*, *Lachnospira* and *Butyricimonas* from MR analysis. **(a)** Differences in *Lachnospira* abundance among HC, PD-NC and PDD (Kruskal-Wallis test); the receiver operating characteristic (ROC) of *Lachnospira* (compare with healthy control); the confusion matrix of *Lachnospira* discriminating abilities (compare with healthy control). Scatter plot of the association between *Lachnospira* and Mini-Mental State Examination scores (MMSE). **(b)** Differences in *Roseburia* and *Butyricimonas* abundance among HC, PD-NC and PDD (Kruskal-Wallis test). **p*<0.05 and ***p*<0.01.

SUPPLEMENTARY FIGURE 3 MR analysis on the causal effect of the metabolite and immune on PDD. **(a,b)** Circos plot shows the association between metabolite, immune cells and PDD via five methods with *p*-values <0.05. **(c)** bidirectional and mediating MR analysis among *Subdoligranulum*, mediator (maltose to sucrose ratio) and PDD.
