# Supplementary figures and images for: Effects of gut microbiota on cognitive impairment in Parkinson’s disease: a comprehensive Mendelian randomization and case–control study
Source: Front Microbiol. 2025 Aug 25;16:1620449. doi: 10.3389/fmicb.2025.1620449 (PMC12415775; doi:10.3389/fmicb.2025.1620449)

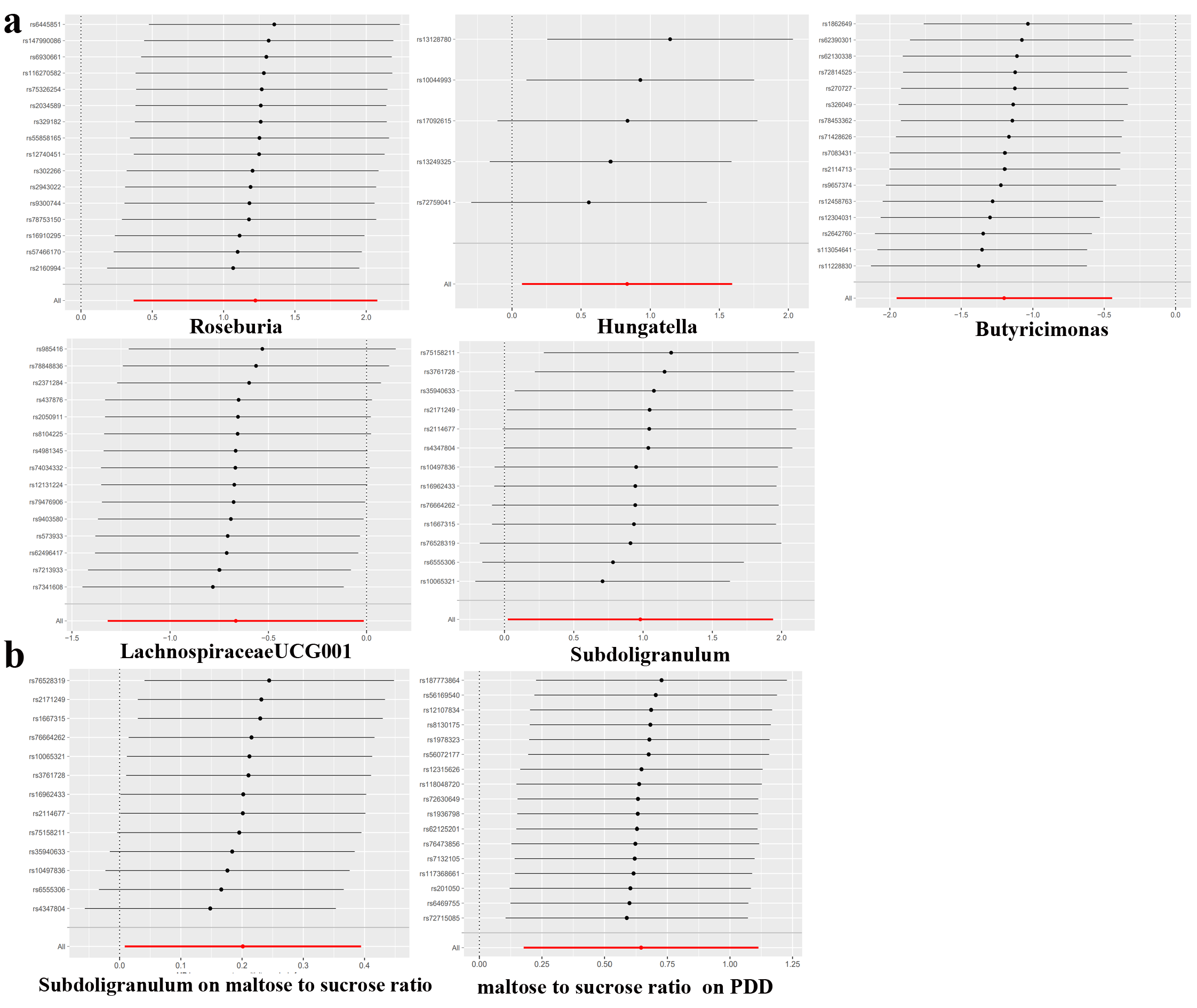

Supplement: SUPPLEMENTARY FIGURE 1 — (a,b) MR leave-one-out sensitivity for five gut microbiota on PD, Subdoligranulum on maltose to sucrose ratio and maltose to sucrose ratio on PDD. [file Image_1.tif]

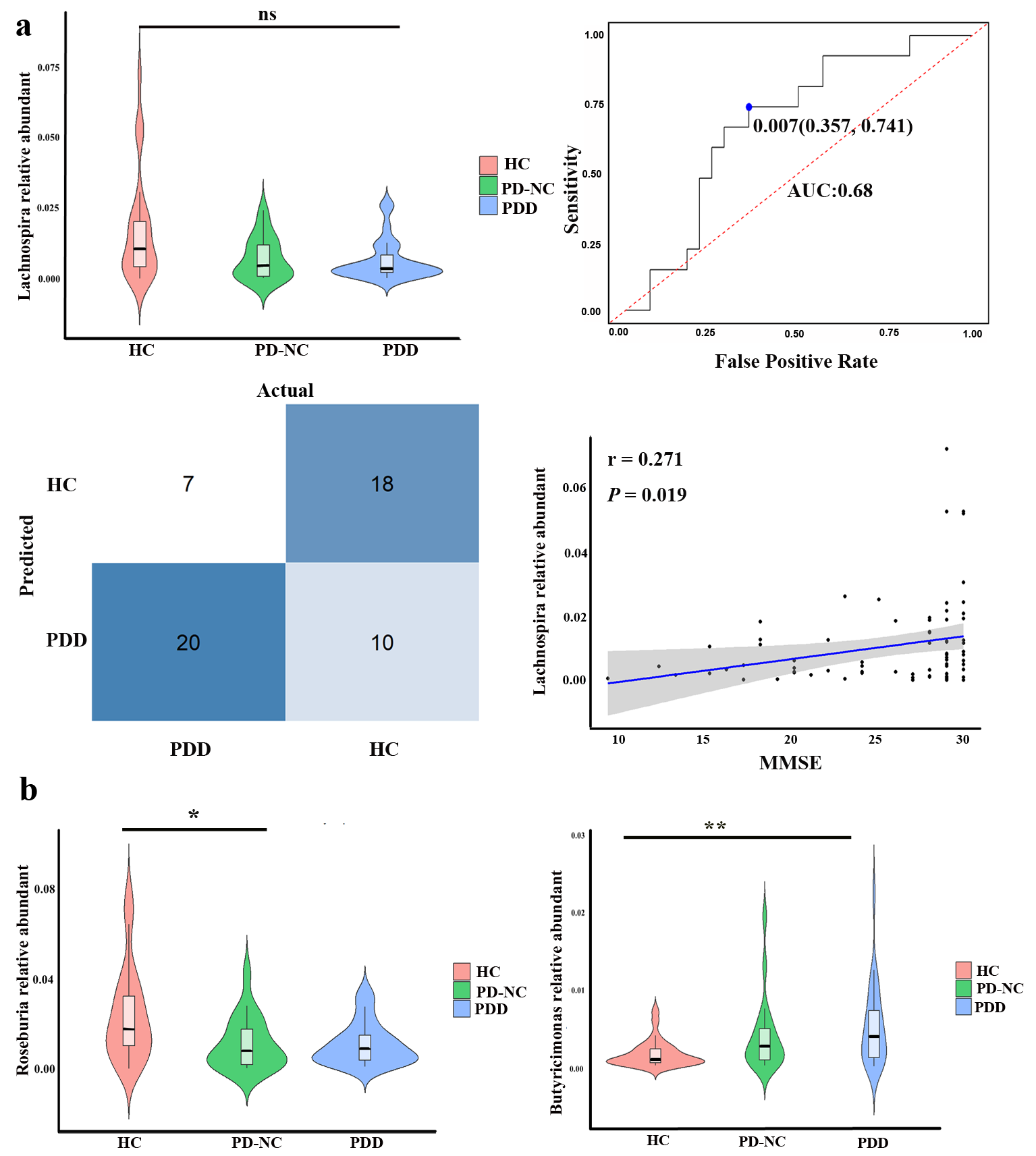

Supplement: SUPPLEMENTARY FIGURE 2 — Clinical evaluation of Roseburia, Lachnospira and Butyricimonas from MR analysis. (a) Differences in Lachnospira abundance among HC, PD-NC and PDD (Kruskal-Wallis test); the receiver operating characteristic (ROC) of Lachnospira (compare with healthy control); the confusion matrix of Lachnospira discriminating abilities (compare with healthy control). Scatter plot of the association between Lachnospira and Mini-Mental State Examination scores (MMSE). (b) Differences in Roseburia and Butyricimonas abundance among HC, PD-NC and PDD (Kruskal-Wallis test). *p<0.05 and **p<0.01. [file Image_2.tif]

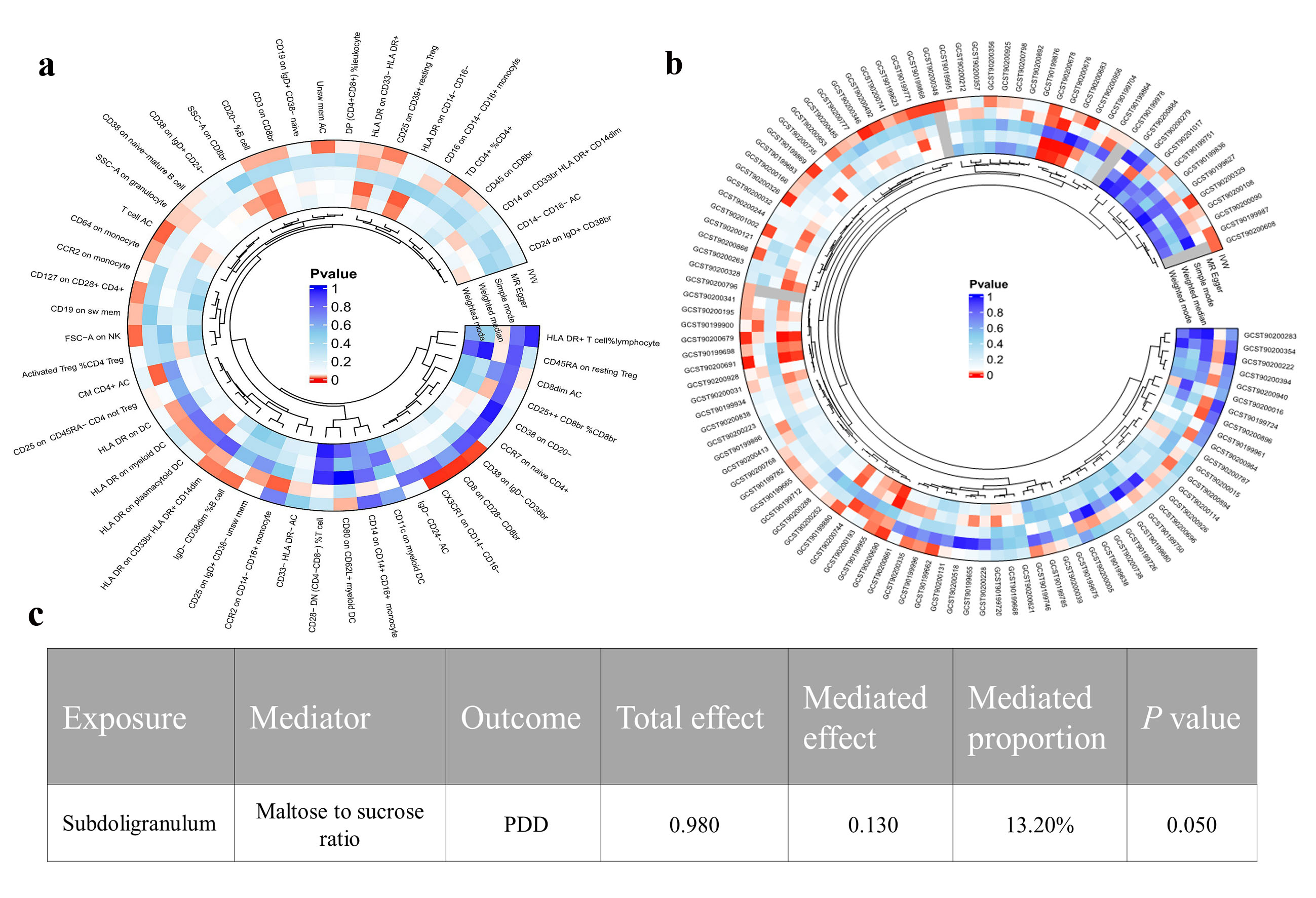

Supplement: SUPPLEMENTARY FIGURE 3 — MR analysis on the causal effect of the metabolite and immune on PDD. (a,b) Circos plot shows the association between metabolite, immune cells and PDD via five methods with p-values <0.05. (c) bidirectional and mediating MR analysis among Subdoligranulum, mediator (maltose to sucrose ratio) and PDD. [file Image_3.jpeg]
